# High frequency oscillatory ventilation compared with conventional mechanical ventilation in adult respiratory distress syndrome: a randomized controlled trial [ISRCTN24242669]

**DOI:** 10.1186/cc3737

**Published:** 2005-06-21

**Authors:** Casper W Bollen, Gijs Th J van Well, Tony Sherry, Richard J Beale, Sanjoy Shah, George Findlay, Mehran Monchi, Jean-Daniel Chiche, Norbert Weiler, Cuno SPM Uiterwaal, Adrianus J van Vught

**Affiliations:** 1 Fellow, Intensive Care, University Medical Centre Utrecht, The Netherlands; 2 Paediatrician, University Medical Centre Utrecht, The Netherlands; 3 Intensivist, St Thomas Hospital, London, UK; 4 Head, Intensive Care, St Thomas Hospital, London, UK; 5 Intensivist, University Hospital of Wales, Cardiff, UK; 6 Intensivist, Hopital Cochin, Paris, France; 7 Intensivist, University Hospital Mainz, Germany; 8 Clinical Epidemiologist, University Medical Centre Utrecht, The Netherlands; 9 Head, Intensive Care University Medical Centre Utrecht, The Netherlands

## Abstract

**Introduction:**

To compare the safety and efficacy of high frequency oscillatory ventilation (HFOV) with conventional mechanical ventilation (CV) for early intervention in adult respiratory distress syndrome (ARDS), a multi-centre randomized trial in four intensive care units was conducted.

**Methods:**

Patients with ARDS were randomized to receive either HFOV or CV. In both treatment arms a priority was given to maintain lung volume while minimizing peak pressures. CV ventilation strategy was aimed at reducing tidal volumes. In the HFOV group, an open lung strategy was used. Respiratory and circulatory parameters were recorded and clinical outcome was determined at 30 days of follow up.

**Results:**

The study was prematurely stopped. Thirty-seven patients received HFOV and 24 patients CV (average APACHE II score 21 and 20, oxygenation index 25 and 18 and duration of mechanical ventilation prior to randomization 2.1 and 1.5 days, respectively). There were no statistically significant differences in survival without supplemental oxygen or on ventilator, mortality, therapy failure, or crossover. Adjustment by *a priori *defined baseline characteristics showed an odds ratio of 0.80 (95% CI 0.22–2.97) for survival without oxygen or on ventilator, and an odds ratio for mortality of 1.15 (95% CI 0.43–3.10) for HFOV compared with CV. The response of the oxygenation index (OI) to treatment did not differentiate between survival and death. In the HFOV group the OI response was significantly higher than in the CV group between the first and the second day. A *post hoc *analysis suggested that there was a relatively better treatment effect of HFOV compared with CV in patients with a higher baseline OI.

**Conclusion:**

No significant differences were observed, but this trial only had power to detect major differences in survival without oxygen or on ventilator. In patients with ARDS and higher baseline OI, however, there might be a treatment benefit of HFOV over CV. More research is needed to establish the efficacy of HFOV in the treatment of ARDS. We suggest that future studies are designed to allow for informative analysis in patients with higher OI.

## Introduction

Mechanical ventilation of patients with adult respiratory distress syndrome (ARDS) may cause lung injury and, subsequently, multi-organ failure [1]. Multi-organ failure is a major cause of death in ARDS [2]. In particular, repetitive opening and closure of alveoli with significant shear forces exerted to the alveolar walls and over-distension of alveoli and small airways are thought to be main factors leading to ventilator induced lung injury. Lung protective ventilation strategies with low tidal volumes and high end-expiratory pressures are used to prevent ventilator induced lung injury [3]. In high frequency oscillatory ventilation (HFOV), extremely small tidal volumes are combined with a high mean airway pressure to prevent atelectasis and at the same time limit peak inspiratory pressures. HFOV is suggested, by some, to be the theoretically most optimal form of lung protective ventilation [4]. The role of HFOV in ARDS, however, has to be established yet.

Most studies comparing HFOV with conventional mechanical ventilation (CV) have been performed in premature neonatal patients [5]. The routine use of HFOV as an elective treatment in premature neonates with respiratory distress is equivocal. In a recent paper we have argued that improvements in CV strategies have diminished the relative benefit of HFOV [6]. There is much less evidence in adult and paediatric patients. Three non-randomized prospective trials and no more than two randomized controlled trials in patients with ARDS have been published to establish the safety and efficacy of HFOV [7-11]. In these trials, the oxygenation index (OI), a cost benefit ratio of inspired oxygen times airway pressure divided by arterial oxygen pressure (OI = FiO2 × MAP × 100)/paO2), was an important predictor of mortality.

We performed a randomized controlled trial designed to test the safety and efficacy of HFOV as a primary mode of ventilation in ARDS patients compared with CV. This study was prematurely terminated because of a low inclusion rate and the completion of a similar trial [7]. We compared survival without supplemental oxygen or on ventilator, mortality, therapy failure and crossover.

## Materials and methods

Between October 1997 and March 2001 61 patients were enrolled in a randomized controlled trial comparing HFOV with CV in patients with ARDS to detect differences in mortality, therapy failure and ventilatory support at 30 days. This study was conducted in intensive care units in London, Cardiff, Paris and Mainz. Patients with ARDS and a bodyweight greater than 35 kg were randomized to receive either HFOV or CV. ARDS was defined as the pressure of arterial oxygen divided by the fraction of inspired oxygen (paO2/FiO2) < 200 mmHg, radiographic evidence of bilateral infiltrates on chest X-ray and no evidence of atrial hypertension. Patients with a non-pulmonary terminal disease, severe chronic obstructive pulmonary disease or asthma and grade 3 or 4 air-leak were excluded. Patients with FiO2 > 0.80 for 48 h or more than 10 days of mechanical ventilation before meeting the entry criteria were excluded as well. Randomization was by a sequentially numbered computerized randomization algorithm. The allocation to treatment was concealed until study entry. This study was approved by the ethical committee board of all participating institutions and was in compliance with the Helsinki Declaration. Informed consent was obtained from next of kin of patients prior to study entry.

The general physiological targets for the two ventilator arms were similar. The oxygenation goal was to maintain an O2 saturation ≥ 88% or paO2 > 60 mmHg with a FiO2 < 0.6. The ventilatory goal was to establish an arterial pH > 7.20 and a HCO3 > 19 mmol/l while minimizing peak inspiratory pressures irrespectively of arterial carbon dioxide pressure (paCO2). The priority in both treatment arms was to maintain lung volume by first weaning FiO2 to < 0.60 after which mean airway pressure and FiO2 were given equal priority for reduction. Patients were crossed over to the alternative ventilator in case of therapy failure: intractable hypotension despite maximum support (RR mean < 60 mmHg for > 4 h or < 50 mmHg for > 1 h); intractable respiratory acidosis (pH 7.20 at HCO3 > 19 mmol/l for > 6 h); oxygenation failure (rising OI of more than two times since study entry or OI > 42 after 48 h; OI = (FiO2 × MAP × 100)/paO2)); and grade 4 air leak (air leak with multiple recurrences (> 4); air leak requiring more than two chest tubes per hemithorax; air leak continuing longer than 120 h; or pneumopericardium or pneumoperitoneum). Patients could be withdrawn from the study treatment for the following reasons: withdrawal of consent; weaned from mechanical ventilation; death or treatment failure after crossover.

In the CV treated group, patients were treated with time cycled pressure controlled ventilation. Respiratory rate to achieve low tidal volumes was free up to 60/minute. Maximum peak inspiratory pressure was limited to 40 cmH2O. To minimize the inspiratory pressures, an arterial pH > 7.20 was acceptable irrespectively of the level of paCO2. Positive end-expiratory pressure was advocated up to 15 cmH2O. An inspiratory:expiratory ratio up to 2:1 could be used to achieve adequate oxygenation. Otherwise, the patient was crossed over to HFOV as indicated above. More detailed ventilation procedures and methods of weaning were according to standard protocols of the investigating centres.

Patients in the HFOV group were ventilated with the SensorMedics 3100B ventilator (SensorMedics, Bilthoven, the Netherlands). A high lung volume strategy was used as has been previously described [12]. HFOV was started with continuous distending pressure (CDP) at 5 cm H2O higher than mean airway pressure (MAP) on CV and then adjusted to achieve and maintain optimal lung volume. Therefore, initially, CDP was increased until an O2 saturation > 95% was achieved. CDP was not decreased until FiO2 < 0.60 was feasible applying the general physiological targets mentioned earlier. Pulmonary inflation was checked by chest X-rays if increasing CDP did not result in O2 saturation > 88%. Frequency was initially set at 5 Hz with an inspiratory time of 33%. Delta P was adjusted according to paCO2 and chest wall vibrations. If ventilation did not improve despite a maximum Delta P, the frequency could be lowered. Weaning was instigated if paO2 > 60 mmHg at FiO2 < 0.40 and suction was well tolerated by decreasing Delta P and CDP to continuous positive airway pressure level. Ventilator weaning was continued on CV according to the standard protocol of the unit.

### Measurements

Assessment of the principal outcomes and repeated measurements was not blinded. The principal outcomes consisted of: cumulative survival without mechanical ventilation or oxygen dependency at 30 days; mortality at 30 days; therapy failure; crossover rate; and persisting pulmonary problems defined as oxygen dependency or still being on a ventilator at 30 days. Data collection began one hour following randomization for the conventionally treated patients and at the initiation of HFOV for the HFOV treated patients. The time period on CV prior to the study, ET tube length and diameter, air leak score, Acute Physiologic and Chronic Health Evaluation (APACHE) II score at admission, arterial blood gases, ventilator settings and cardiovascular measurements were recorded. Arterial blood gases, ventilator settings, heart rate, blood pressure and cardiac output, if available, were registered after study entry or crossover and every eight hours for four days on the assigned ventilator. Ventilator settings and blood gases were recorded for every change of ventilator settings during the first three days of treatment.

### Statistical analysis

In analyses of primary outcomes, the intention to treat principle was used. Based on a projected survival without mechanical ventilation or oxygen dependency in the control group of 25%, an increase to 51% in the HFOV group would be detectable with 106 patients (alpha of 0.05, power of 0.80) [9]. Univariate logistic regression analysis was used to calculate differences in 30 day survival without mechanical ventilation or oxygen dependency, mortality, crossover, therapy failure and incidence of supplemental oxygen dependency or mechanical ventilation at 30 days. Cox proportional hazard analysis was conducted to detect differences in mortality. The proportionality assumption was graphically tested using log minus log plots. Multivariate logistic regression and Cox proportional hazard analysis for mortality were used to adjust in case of post-randomization differences in *a priori *defined pre-treatment conditions (dummy variables for study site, OI, ventilatory index (ventilatory index = (peak inspiratory pressure (mmHg) × respiratory rate × paCO2 (mmHg))/1000), APACHE II score, age and weight). Furthermore, we looked at the relation between the OI response and mortality. Average values and standard errors of respiratory and circulatory parameters were calculated for days 1, 2, 3, and 4 of the study. Significant differences between treatment groups were tested by a general linear mixed model analysis. P-values were calculated 2-sided. All analyses were conducted using SPSS 12.0.1 for Windows software (SPSS Inc., Chicago, Illinois, U.S.).

## Results

The study was stopped prematurely after inclusion of 61 patients because of a low inclusion rate and the completion of another trial comparing HFOV with CV in patients with ARDS [7]. Of the 61 patients, 37 were randomized to receive HFOV and 24 to receive CV. Follow up time to 30 days was incomplete in seven patients (five HFOV and two CV).

The baseline OI at study entry was higher in the HFOV group than in the CV group, (25 versus 18; Table 1). Patients were comparable for age and APACHE II score. The youngest patient was 17 years and the oldest patient was 77 years. The female:male ratio was lower in the HFOV group than in the CV group (0.24 versus 0.42). The majority of patients (80%) were diagnosed with sepsis or pneumonia. Prior to randomization, patients were ventilated with an average tidal volume of 9.3 ml/kg ideal bodyweight in the HFOV group and 8.4 ml/kg ideal bodyweight in the CV group. (Ideal body weight was calculated as: males, weight = 50 + 0.91 × (height in centimetres – 152.4); females, weight = 45 + 0.91 × (height in centimetres – 152.4)). Peak inspiratory pressures were comparable for both treatment groups. In one case, the limitation of 40 mmHg for peak inspiratory pressures was violated in the CV group. There were no major differences between treatment groups in mean airway pressures or peak end-expiratory pressures. Blood gas results prior to randomization showed a lower arterial oxygen saturation and paO2 in the HFOV group compared with the CV group.

**Figure 1:**
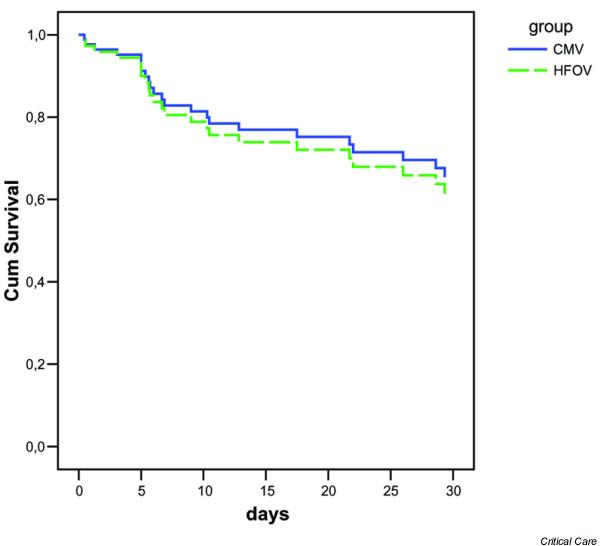
Cumulative mortality incidence for high frequency oscillatory ventilation (HFOV) versus conventional mechanical ventilation (CV). Curves are estimates of cumulative risk corrected for study site, baseline oxygenation index and ventilatory index, APACHE II score, age and weight.

The primary outcomes are presented in Table 2. There was no difference in cumulative survival without oxygen dependency or still on mechanical ventilation at 30 days between HFOV and CV. Mortality at 30 days did not differ significantly between HFOV and CV. An important cause of death was withdrawal of treatment (10 cases in 24 deaths). None of the deaths were directly related to the assigned therapy. Figure 1 shows a nearly identical cumulative survival of the HFOV group and the CV group corrected for the baseline covariates; study site, OI, ventilatory index, APACHE II score, age and weight. The survival curves of the duration of ventilation were virtually identical for the HFOV group and the CV group (data not shown). The median duration of ventilation was 20 days (± 6 SD) for HFOV and 18 days (± 5 SD) in the CV treatment group.

Treatment failure occurred in 10 patients (27%) in the HFOV group compared with five patients (21%) in the CV group. Seven patients (19%) treated with HFOV crossed over to CV; in the CV group four patients (17%) were switched to HFOV. Of the four patients that crossed over in the CV group, two patients died and one patient was on supplemental oxygen therapy at 30 days. In the HFOV group, five patients that crossed over died and two patients were still on ventilator or needed extra oxygen. The occurrence of being on oxygen or mechanical ventilation at 30 days in survivors was equal between HFOV and CV.

Ventilatory settings and blood gas results at days 1, 2, 3 and 4 of the study are shown in Table 3. Patients with HFOV were ventilated with higher mean airway pressures than patients on CV (p = 0.03). FiO2 was also higher in the HFOV group compared with the CV group. This difference between the treatment groups was not significant (p = 0.33). Results of blood gases were comparable between the two treatment groups including all patients. Patients that crossed over in the CMV group had significantly lower pH than patients who did not cross over in the CMV group (p = 0.02). This difference, however, was not found between patients who did and did not cross over in the HFOV group (p = 0.56). The OI, on the other hand, was higher in both patients that crossed over in the CMV group and patients that crossed over in the HFOV group compared with patients that did not cross over (p = 0.07 and p = 0.05, respectively).

Systolic arterial blood pressure and mean arterial blood pressure were higher in the HFOV treated patients compared with CV treated patients (p = 0.06 versus p = 0.07). Cardiac output was comparable between the two treatment groups (data not shown).

**Figure 2:**
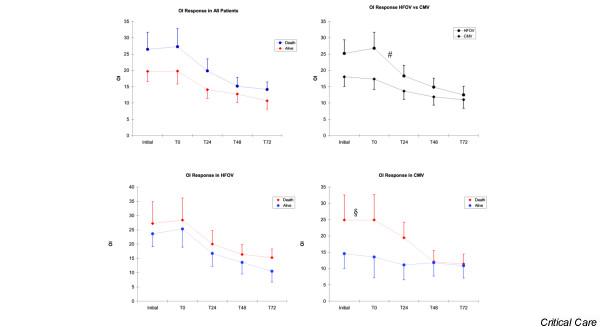
Oxygenation index (OI) in survivors versus non-survivors and high frequency oscillatory ventilation (HFOV) versus conventional mechanical ventilation (CV). OIs are represented by diamonds as means with bars as 95% confidence intervals (CI). Reported p-values for baseline OI are corrected for study site, ventilatory index, APACHE II score, age and weight. The baseline OI did not significantly predict mortality in all patients or in HFOV (p = 0.06 and p = 0.41, respectively). ^§^Baseline OI was significantly different between survivors and non-survivors in the CV group (p = 0.04). Significant differences between OI responses were calculated by linear mixed model analyses. ^#^Significant difference in OI response between HFOV and CV (p = < 0.01). OI response did not differentiate between survivors and non-survivors in all patients or in CV and HFOV separately (p = 0.28, p = 0.12 and p = 0.95, respectively).

The OI response in all patients treated with either HFOV or CV did not differ significantly between survivors and non-survivors (Figure 2). The OI response from day 1 to day 2 was significantly larger in HFOV than in CV treated patients (p < 0.01). Within treatment groups there was a significant difference in initial OI between survivors and non-survivors in CV treated patients, but OI response to treatment did not differentiate between survivors and non-survivors in CV treated patients. In the HFOV treated patients there was no difference in the baseline OI, nor was there a difference in OI response between survivors and non-survivors.

**Figure 3:**
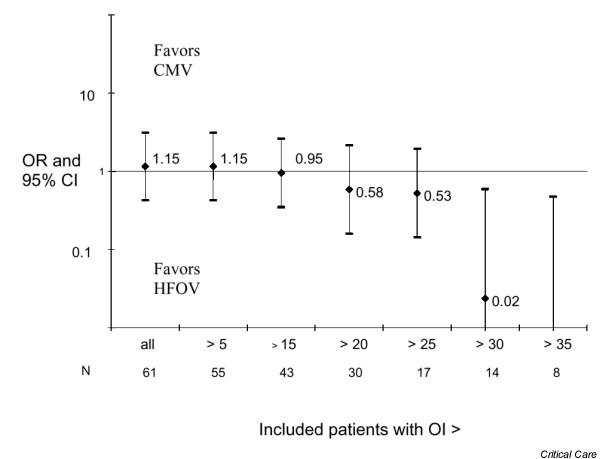
*Post hoc *analysis of the treatment effect on mortality relative to baseline oxygenation index (OI). On the y-axis the odds ratio of mortality (OR) adjusted for study site, OI, ventilatory index, APACHE II score, age and weight is presented by diamonds and 95% confidence intervals by bars. On the x-axis the different analyses are depicted including patients with increasing levels of initial OI at study entry. N denotes the number of patients in each subgroup. CI, confidence interval; CMV, conventional mechanical ventilation; HFOV, high frequency oscillatory ventilation.

The results of a *post hoc *analysis are shown in Figure 3. Adjusted odds ratios for mortality were calculated for samples of the study population including patients with progressively higher baseline OI prior to randomization. This suggested that, in patients with a higher baseline OI, the effect of treatment with HFOV was relatively better compared with CV. OI was evaluated as an interaction term in a Cox Proportional Hazard model with treatment, age and OI as explanatory variables. The likelihood ratio test comparing the reduced (no-interaction) with the full (interaction) model showed a p-value of 0.048.

## Discussion

No significant differences between HFOV and CV were observed, but this trial only had power to detect major differences in mortality or survival without oxygen dependency or on ventilator. Furthermore, 11 of 61 patients were crossed over to a different treatment arm; this also diminished the power to detect potential treatment differences. A *post hoc *analysis, however, suggested that in patients with a higher baseline OI, HFOV may be more effective than CV.

This trial was stopped because of a low inclusion rate and the completion of another similar trial [7]. The low inclusion rate was not because of competing trials but probably due to the limited number of investigators (four centres compared with nine centres in the study by Derdak *et al*.). The number of patients included in the two treatment arms differed considerably. This misbalance was due to stopping the trial early. There were no protocol violations. Furthermore, baseline OI at study entry was higher in the HFOV group than in the CV group. The OI has been recognized as an important prognostic determinant of mortality [13].

HFOV was started early in the course of ARDS. Patients were ventilated on HFOV according to the open lung concept. This resulted in significantly higher mean airway pressures compared with CV ventilated patients. This mainly determined the higher OI in the HFOV group during the first days. FiO2 and paO2 values were similar between HFOV and CV patients. Potential theoretical risks of HFOV therapy, overdistension of the pulmonary system leading to barotrauma or cardiovascular compromise, packing of mucus leading to ineffective ventilation or blocking of the endotracheal tube were not encountered. None of the HFOV ventilated patients developed necrotizing tracheobronchitis.

Patients in the CV group were ventilated following a lung protective strategy targeted to minimizing tidal volumes. The tidal volumes per kg ideal bodyweight that were used in this study were higher than tidal volumes used in studies of lung protective ventilation strategies [14]. On the other hand, tidal volumes in our study were significantly lower than tidal volumes that were found to be harmful in those studies. Peak inspiratory pressures were limited to 40 cmH2O in the CV group. This restriction was violated in only one case. Nine patients were ventilated with pressures above 35 cmH2O. Furthermore, the overall mortality and survival without mechanical ventilation or oxygen dependency at 30 days did not suggest that the ventilation treatment in the CV group was suboptimal.

The OI represents the pressure and oxygen cost for oxygenation. It has been regarded as a marker of lung injury and prognostic indicator of treatment success [15]. In CV treated patients there was a significant difference in baseline OI between survivors and non-survivors. Baseline OI did not, however, differentiate between survivors and non-survivors in HFOV treated patients. Although in some studies OI response to treatment was a predictor of outcome [7,9], we could not reproduce this relation. A possible explanation could be that fewer numbers of patients were included in our analysis. Also, we used a different time window; we compared OI on a daily basis whereas in a study by Derdak *et al*. [7] OI was compared every 4 h. In that study, OI response was maximally different at 16 h [7]. In our study, OI response only differed significantly between HFOV and CV treated patients. This difference for the most part could be explained by the higher mean airway pressures used in the HFOV group.

A *post hoc *analysis suggested that baseline OI could be an important effect modifier of the relative treatment effect of HFOV compared with CV. We hypothesize that within the pressure-ventilation curve there is a safe window between under-inflation with atelectasis and shear stress and over-inflation with barotrauma [4,16]. In patients with ARDS with higher OI, this safe window possibly becomes too small for CV to prevent ventilator induced lung injury. This concept is supported by animal experiments where addition of positive end-expiratory pressure (PEEP) resulted in additional over-inflation contributing to ventilator-induced lung injury [17]. The combination of high levels of PEEP and over-distension are directly reflected in the OI. HFOV seemed to offer an advantage over CV only in patients with a higher initial OI. This is in accordance with observational studies that showed that better survival rates in more severe ARDS with higher OI was associated with HFOV treatment [11,18]. In fact, HFOV has been recommended in patients who require high mean airway pressure and FiO2 exceeding 60% corresponding to an OI > 20 when paO2 = 60 mmHg [12]. Because these findings result from a *post hoc *analysis, however, they can only be regarded as hypothesis generating still to be confirmed.

Previous trials did not show a significant difference in mortality in patients with ARDS between HFOV and CV [19]. In our trial, mortality in the HFOV group was similar to mortality reported in the previous trials, but mortality in the CV group was considerably less, in accordance with the imbalance in prognostic indicators at baseline.

More evidence is needed to confirm a beneficial effect of HFOV over CV in the treatment of ARDS. Our results and those from previous trials seem promising but could depend on other criteria to select patients with ARDS that benefit from HFOV compared with CV. One of these criteria could be OI. Therefore, we believe that in future research comparing HFOV with CV as early treatment of ARDS, it is important to focus on patients with higher levels of baseline OI. As treatment differences will be smaller than our prior estimate was, larger trials are needed. We do not think that OI response can be used as an alternative outcome measurement for treatment success or failure.

## Conclusion

In this study, we were not able to find significant differences in efficacy or safety between HFOV and CV as early treatment of ARDS. A *post hoc *analysis suggested that HFOV could prevent mortality compared with CV in patients with a higher baseline OI. Therefore, it is important in future studies to enable informative analysis of patients with higher baseline OI. To achieve sufficient power to detect possible important treatment differences in subgroups of patients with higher OI, larger multi-centre trials are warranted.

## Key messages

• This study was not powered to show significant differences in efficacy or safety between HFOV and CV as early treatment of ARDS.

• However, a post hoc analysis suggested a better treatment effect of HFOV compared with CV in patients with higher baseline OI.

• Future studies should be designed to allow for informative analysis in patients with higher OI.

## Abbreviations

ARDS = adult respiratory distress syndrome; CDP = continuous distending pressure; CI = confidence interval; CV = conventional mechanical ventilation; FiO2 = fraction of inspired oxygen; HFOV = high frequency oscillatory ventilation; MAP = mean airway pressure; OI = oxygenation index; OR = odds ratio; paCO2 = pressure of arterial carbon dioxide; paO2 = pressure of arterial oxygen; PEEP = positive end-expiratory pressure; SaO2 = arterial oxygen saturation.

## Competing interests

Supported in part by SensorMedics Corporation, which also provided use of the 3100B high-frequency oscillatory ventilators. None of the study investigators have a financial interest in SensorMedics Corporation. The authors declare that they have no competing interests.

## Authors' contributions

AJvV initiated the study, participated in its design and coordination and helped to draft the manuscript. CWB, CSPMU and GTJvW performed the statistical analyses and wrote the manuscript. TS, RJB, SS, GF, MM, JC and NW participated in its design and conducted the study. All authors read and approved the final manuscript.

## Figures and Tables

**Table 1:** Patient characteristics at study entry

	HFOV	CV
N	37	24
Female:male ratio	9/28 (24%)	10/14 (42%)
Mean age (years)	81.0 ± 20.5	81.7 ± 12.5
Weight	50.7 ± 17.4	55.4 ± 12.8
APACHE II score	21.1 ± 7.6	20.1 ± 9.3
Diagnosis (%)		
Trauma	1 (3)	2 (9)
Sepsis	25 (68)	13 (57)
Pneumonia	8 (22)	3 (13)
Other	3 (8)	5 (22)
Site (%)		
United Kingdom	24 (65)	15 (63)
France	5 (21)	7 (19)
Germany	4 (17)	6 (16.2)
Ventilation time prior to study (days)	2.1 ± 2.6	1.5 ± 1.8
Oxygenation index	25.2 ± 13.0	18.0 ± 7.4
Ventilatory index	33.8 ± 20.4	30.3 ± 12.5
Respiratory rate (per min)	18.1 ± 4.1	17.8 ± 4.6
Tidal volume(ml)	618.4 ± 142.6	549.7 ± 130
Tidal volume per ideal bodyweight (ml/kg)	9.3 ± 2.2	8.4 ± 2.0
Peak inspiratory pressure (cmH2O)	33.1 ± 6.8	32.3 ± 5.4
Positive end-expiratory pressure (cmH2O)	13.9 ± 3.8	12.9 ± 3.2
Mean airway pressure (cmH2O)	21.5 ± 5.4	21.0 ± 5.1
FiO2	0.84 ± 0.19	0.76 ± 0.19
pH	7.3 ± 0.13	7.3 ± 0.11
paCO2 (mmHg)	53.5 ± 17.3	52.2 ± 11.9
paO2 (mmHg)	80.8 ± 24.1	93.3 ± 24.5
SaO2 (percentage)	90.8 ± 6.4	94.3 ± 3.1
Heart rate	109.8 ± 23.7	111.2 ± 29.5
Mean arterial pressure (cmH2O)	75.3 ± 13.1	72.2 ± 14.1
Central venous pressure (cmH2O)	13.5 ± 4.2	13.8 ± 4.9

Values are presented as means with standard deviations. APACHE II, Acute Physiologic and Chronic Health Evaluation II; CV, conventional mechanical ventilation; FiO2, fraction of inspired oxygen; HFOV, high frequency oscillatory ventilation; OI, oxygenation index; paO2, pressure of arterial oxygen, paCO2, pressure of arterial carbon dioxide; SaO2, arterial oxygen saturation.

**Table 2:** Primary outcomes

				Unadjusted		Adjusted	
	HFOV	CV	p-value	OR	95% CI	OR	95% CI

N	37	24					
Survival without supplemental oxygen or on ventilator	12 (32%)	9 (38%)	0.79	0.80	0.27–2.53	0.80	0.22–2.97
Mortality	16 (43%)	8 (33%)	0.59	1.52	0.45–2.59	1.15	0.43–3.10
Circulatory failure	6	2					
Cardiac arrhythmia	3	1					
Brain death	0	2					
Withdrawal of life support	7	3					
Therapy failure	10 (27%)	5 (21%)	0.76	1.41	0.41–4.78	1.35	0.35–5.22
Hypotension	4	1					
Acidosis	1	1					
Oxygenation	4	2					
Air leak	1	1					
Cross-over	7 (19%)	4 (17%)	0.82	1.17	0.30–4.51	0.62	0.12–3.19
Supplemental oxygen or on ventilator at 30 days	9 (24%)	7 (29%)	0.96	0.96	0.26–3.58	0.67	0.12–3.84

Values between brackets are percentages of N (number of patients included in the analyses) except for CLD (Chronic Lung Disease) that has the number of survivors in the denominator. CI, confidence interval; OR, odds ratio unadjusted and adjusted for study site, OI, ventilatory index, APACHE II score, age and weight.

**Table 3:** Ventilatory conditions

	HFOV		CV	
Cross-over	No (30)	Yes (7)	No (20)	Yes (4)

Day 1	N = 28	N = 7 (7 HFOV)	N = 19	N = 4 (4 CV)

Peak inspiratory pressure (cmH2O)			32 ± 4.2	35 ± 6.9
Positive end-expiratory pressure (cmH2O)			14 ± 2.1	12 ± 4.5
Mean airway pressure (cmH2O)	30 ± 5.6^a^	32 ± 6.3^a^	22 ± 3.2	22 ± 6.1
Tidal volume per ideal bodyweight (ml/kg)			9 ± 1.7	8 ± 0.7
Frequency (HFOV, Hz; CV, breaths/min)	5 ± 0.5	5 ± 0.9	17.3 ± 3	17.3 ± 6
Delta P (cmH2O)	63 ± 14	70 ± 12.1		
FiO2	0.78 ± 0.19	0.82 ± 0.12	0.68 ± 0.12	0.78 ± 0.21
pH	7.32 ± 0.08	7.31 ± 0.11	7.34 ± 0.08	7.22 ± 0.07^b^
pCO2 (mmHg)	49 ± 11.3	57 ± 13	48 ± 9	52 ± 15.8
pO2 (mmHg)	126 ± 79.2	93 ± 37.1	98 ± 26.6	99 ± 25
SaO2 (percentage)	95 ± 3	90 ± 10.7	96 ± 2.4	94 ± 4.5
Oxygenation index	26 ± 16	31 ± 8.3^c^	17 ± 7.5	19 ± 11.2^c^
Day 2	N = 27	N = 7 (6 HFOV)	N = 19	N = 4 (2 CV)
Peak inspiratory pressure (cmH2O)	25 ± 6.7	36 ± 7.2	31 ± 4.5	30 ± 2.6
Positive end-expiratory pressure (cmH2O)	11 ± 1.2	15 ± 1.9	14 ± 2.7	12 ± 4.7
Mean airway pressure (cmH2O)	28 ± 6.7^a^	29 ± 4.3^a^	21 ± 2.3	22 ± 9.1
Tidal volume per ideal bodyweight (ml/kg)	9 ± 1.6	10 ± 1.9	8 ± 1.6	8 ± 1
Frequency (HFOV, Hz; CV, breaths/min)	5.0 ± 0.4	4.8 ± 1.1	17.4 ± 2.6	17.2 ± 1.2
Delta P (cmH2O)	64 ± 14.5	73 ± 14.8		70 ± 13.8
FiO2	0.55 ± 0.17	0.57 ± 0.14	0.53 ± 0.12	0.76 ± 0.20
pH	7.36 ± 0.07	7.35 ± 0.04	7.38 ± 0.06	7.22 ± 0.08^b^
pCO2 (mmHg)	45 ± 9	51 ± 8.9	46 ± 8.3	53 ± 8.5
pO2 (mmHg)	96 ± 21	83 ± 12.4	100 ± 27	87 ± 41.8
SaO2 (percentage)	95 ± 2.1	94 ± 1.9	96 ± 1.8	87 ± 16.1
Oxygenation index	17 ± 10.2	21 ± 8.2^c^	12 ± 3.6	22 ± 10.5^c^
Day 3	N = 23	N = 7 (4 HFOV)	N = 19	N = 4 (2 CV)
Peak inspiratory pressure (cmH2O)	21 ± 3.1	32 ± 12	30 ± 4	27 ± 6
Positive end-expiratory pressure (cmH2O)	9 ± 3	10 ± 4.3	13 ± 2.8	11 ± 5.7
Mean airway pressure (cmH2O)	23 ± 7.1^a^	25 ± 6.9^a^	20 ± 2.8	24 ± 2.3
Tidal volume per ideal bodyweight (ml/kg)	9 ± 1.5	9 ± 3.5	9 ± 1.6	7 ± 1.6
Frequency (HFOV, Hz; CV, breaths/min)	5.0 ± 0.4	4.6 ± 0.5	18.8 ± 6.5	19.9 ± 5.8
Delta P (cmH2O)	66 ± 12.4	66 ± 19.1		67 ± 0.7
FiO2	0.46 ± 0.13	0.55 ± 0.15	0.46 ± 0.11	0.65 ± 0.26
pH	7.39 ± 0.06	7.37 ± 0.06	7.39 ± 0.06	7.33 ± 0.1^b^
pCO2 (mmHg)	45 ± 10.4	47 ± 12.9	48 ± 9	47 ± 12.6
pO2 (mmHg)	89 ± 19.7	86 ± 46.2	91 ± 13.7	89 ± 22.4
SaO2 (percentage)	94 ± 6.7	89 ± 14.1	96 ± 1.9	95 ± 2.4
Oxygenation index	14 ± 7.2	19 ± 9.3^c^	11 ± 3.7	20 ± 12.3^c^
Day 4	N = 22	N = 7 (3 HFOV)	N = 19	N = 2 (0 CV)
Peak inspiratory pressure (cmH2O)	25 ± 8	31 ± 6.9	28 ± 6.9	
Positive end-expiratory pressure (cmH2O)	9 ± 4.6	11 ± 4.2	11 ± 3.2	
Mean airway pressure (cmH2O)	22 ± 7.8^a^	24 ± 6.2^a^	17 ± 5.6	24 ± 3.2
Tidal volume per ideal bodyweight (ml/kg)	10 ± 2.4	7 ± 3.1	8 ± 2.2	
Frequency (HFOV, Hz; CV, breaths/min)	5.0 ± 0.3	4.3 ± 0.6	17.9 ± 5.3	
Delta P (cmH2O)	57 ± 11.4	70 ± 11.8		48 ± 14.8
FiO2	0.45 ± 0.11	0.57 ± 0.18	0.45 ± 0.11	0.51 ± 0.12
pH	7.42 ± 0.14	7.37 ± 0.1	7.43 ± 0.12	7.45 ± 0.06^b^
pCO2 (mmHg)	43 ± 12.3	46 ± 7.5	41 ± 10.3	44 ± 11.1
pO2 (mmHg)	85 ± 22.3	84 ± 30.5	87 ± 27.4	74 ± 23.7
SaO2 (percentage)	89 ± 15.3	90 ± 14.1	89 ± 17.2	84 ± 20
Oxygenation index	12 ± 5.6	18 ± 7.9^c^	10 ± 4.3	19 ± 9.5^c^

The columns represent the treatment allocation. Measurements were made day 1, 2, 3 and 4 of the study. Peak inspiratory pressure, positive end-expiratory pressure and tidal volume per ideal bodyweight were measured in high frequency oscillatory ventilation (HFOV) after crossover to conventional mechanical ventilation (CV). Values are presented as means with standard deviations. ^a^Higher mean airway pressures in HFOV compared with CV (p = 0.03). ^b^Significantly lower pH in patients that cross over in the CV group (p = 0.017). ^c^Higher OI in patients that crossed over compared with patients that did not cross over (p = 0.07 and p = 0.05, respectively). FiO2, fraction of inspired oxygen; paCO2, pressure of arterial carbon dioxide; paO2, pressure of arterial oxygen; SaO2, arterial oxygen saturation.
